# The Public's Risk Information Seeking and Avoidance in China During Early Stages of the COVID-19 Outbreak

**DOI:** 10.3389/fpsyg.2021.649180

**Published:** 2021-03-12

**Authors:** Mei Liu, You Chen, Dan Shi, Tingwu Yan

**Affiliations:** ^1^Department of Business Administration, College of Economics and Management, Huazhong Agricultural University, Wuhan, China; ^2^Department of Agricultural Economics and Management, College of Economics and Management, Huazhong Agricultural University, Wuhan, China

**Keywords:** information seeking, information avoidance, COVID-19, emerging risk, risk perception

## Abstract

This study uses the Planned Risk Information Seeking Model (PRISM) to estimate the public's information seeking and avoidance intentions during the COVID-19 outbreak based on an online sample of 1031 Chinese adults and provides support for the applicability of PRISM framework in the situation of a novel high-level risk. The results indicate that information seeking is primarily directed by informational subjective norms (ISN) and perceived seeking control (PSC), while the main predictors of information avoidance include ISN and attitude toward seeking. Because ISN are the strongest predictor of both information seeking and avoidance, the way the public copes with COVID-19 information may be strongly affected by individuals' social environment. Furthermore, a significant relationship between risk perception and affective risk response is identified. Our results also indicate that people who perceive greater knowledge of COVID-19 are more likely to report greater knowledge insufficiency, which results in less information avoidance.

## Introduction

Since the outbreak of COVID-19 at the end of 2019, it has spread globally, and confirmed cases have surged worldwide with worrisome speed. As of 4:22 pm, CET, February 18, 2021, there have been 109,594,835 of confirmed cases of COVID-19, including 2,424,060 deaths according to the latest data of the World Health Organization (2021), which has declared that COVID-19 is a pandemic, meaning an escalation in both the severity of it and the difficulty of fighting it.

People of all ages lack immunity and are generally susceptible to COVID-19. The main routes of transmission are respiratory droplets and close contact (the World Health Organization, 2020). The novel coronavirus seems to be very “cunning” due to its uncertain origin, new variants of the virus, high covertness, and high transmissibility (Hao et al., 2020), which makes it very difficult to contain the pandemic. The pandemic has greatly affected the economic development and social stability of many countries and has caused psychological panic among most people (Siebenhaar et al., 2020). The global growth contraction for 2020 is estimated at −3.5 percent by the International Monetary Fund (2021), which is the agency's most pessimistic forecast since the Great Depression of the last century.

Research on risk information seeking recognizes that information is essential when a person feel threatened by a potential harm and he/she is unsure of the most effective dependence (Yang et al., 2014). Thus, to a certain extent, whether the pandemic can be controlled in a short time depends on whether people have relevant knowledge (i.e., information) and respond to the measures taken by the government against the pandemic. That is why the WHO has reminded people to pay close attention to the latest news on the COVID-19 outbreak. An increase in risk information and information seeking can lead to positive outcomes by helping the public improve prevention, decrease the risk of infection, reduce the uncertainty, and alleviate the panic (Winkleby et al., 1994; Viswanath and Finnegan, 1996; Yang et al., 2014; Lep et al., 2020). As an important topic of communication studies, risk information seeking has drawn increased attention from health communication scholars, who have focused on its characteristics, antecedents, and outcomes to fully comprehend individuals' risk information seeking and processing since the mid −1990s (Lambert and Loiselle, 2007).

This study employs the Planned Risk Information Seeking Model (PRISM) developed by Kahlor (2010) to focus on the public's COVID-19 risk information seeking intention and its drivers during the outbreak. The model has been used in the situation of general health risks and some specific diseases such as cancer (Hovick et al., 2014a), but few studies (only one paper by Hubner and Hovick, 2020) have adopted the model regarding an infectious disease during its outbreak period. Because of the new coronavirus being quite different from other infectious diseases such as the Zika virus, and its worldwide and sustained social impact (as the WHO officials asserted on May 13, 2020, that new coronavirus will be present for a long time), we conduct this research on it via the PRISM model, which will undoubtedly enrich and expand PRISM-related research. In addition to seeking intention, information avoidance, which deserves more attention suggested by Kahlor (2010), plays an important role in information management in health contexts. Therefore, information avoidance will also be explored according to the PRISM model in the present study to determine its motivators, which has not been explored yet specifically via PRISM, to better understand the public's COVID-19 risk information decision-making behavior.

The COVID-19 pandemic broke out in Wuhan city since December, 2019, people in Wuhan had to cope with the pandemic somewhat earlier than many other folks around the world. Obviously, it is very necessary to investigate and understand their responses to the pandemic, especially their risk information seeking and avoidance intentions to better prevent or control the spread of the pandemic. Thus, at the beginning of March last year, we conducted a questionnaire survey of the Wuhan adults.

## Theoretical Framework

PRISM, which is a generalizable theoretical framework, provides individual-level predictors of the intention to seek information primarily derived from the Risk Information Seeking and Processing model (RISP, Griffin et al., 1999a; Kahlor, 2010) and the Theory of Planned Behavior (TPB; Ajzen, 1991). PRISM is a comprehensive model of the above models, but outperforms the TPB or the RISP alone in explaining information seeking on health risk, which was confirmed by Kahlor (2010)'s research. This model regards risk information seeking as a planned behavior and holds that the intention to seek risk information is guided by the public's perceived risk, affective risk response, perception of knowledge and knowledge insufficiency, and beliefs toward information seeking (Kahlor, 2010).

Risk information seeking can be defined as “more or less effortful attempts to gather information through a variety of mediated and interpersonal channels to achieve personal goals, including those representing various cognitive and affective motivations” (Griffin et al., 2012a), in short, the effort paid to find related information regarding one's personal risk of COVID-19 in this study. It includes both routed seeking (“fairly passive exposure to risk-related information based on media use habits”) and non-routed seeking (“more active efforts to gather risk-related information that go beyond habitual sources”) (Griffin et al., 2012b). Although information or knowledge related to a certain disease is very important and valuable (i.e., contributing to an in-depth understanding of disease symptoms, prevention strategies, or effective cures), people do not always seek it and sometimes spare no effort to avoid it (Turner et al., 2006; Sweeny et al., 2010; Sweeny and Miller, 2012). That is, risk information avoidance is also a very important (and potentially disruptive) behavior in the middle of a pandemic, which can be conceptualized as any behavior aimed at preventing or delaying the acquisition of available but potentially unwanted information (Sweeny et al., 2010). It can be active (e.g., by asking someone not to reveal information, physically leaving a situation to escape from learning information, turning off radios or televisions to avoid hearing about risk-related topics) or passive (e.g., by failing to ask someone a question that would reveal the information) (Sweeny et al., 2010; Barbour et al., 2012). In fact, people frequently avoid risk information seeking when the topic is disturbing (Brashers et al., 2000; Case et al., 2005) or contrary to their belief system (Zillman and Bryant, 1985; Babrow, 2001). In addition, research suggests that people may choose to avoid information because the information might: (a) compel them to give up or change cherished belief, (b) force them to take an action or adopt a behavior that they would rather not undertake, and (c) cause unpleasant emotions or reduce pleasant emotions (Sweeny et al., 2010).

Compared with information seeking, information avoidance has been relatively understudied. And research on this topic by PRISM has not been found, although a few researchers have examined information avoidance using the RIS-P model, when people choose to maintain a degree of information insufficiency because their uncertainty assessment shows that they do not want to obtain more information about the risk (Yang et al., 2011; Yang and Kahlor, 2013). Such studies propose that if an individual feels that he/she has enough information to deal with the risk, it may lead to avoidance (accuracy motivation) or be base-d on two of the other motivations (defensive and impression motivations) from the Heuristic Systematic Model, the latter two motivations not having been tested yet in the RISP (Griffin et al., 2012a). A multi-application analysis of RISP, as applied to health and environmental risks (Dunwoody and Griffin, 2015, table on p.109), found that individuals are more likely to avoid risk information when they realized they already had adequate information about the risk (less insufficiency meant more avoidance—but the opposite regarding seeking), and when they believed that the channels of risk information were biased; avoidance was less likely when people felt social pressures to understand the information (informational subjective norms) and when they were capable of critically seeking and evaluating the information, based on the revised capacity measures used in the latter two studies reported.

Therefore, information seeking and avoidance may be a balancing behavior for the public to attain several objectives (reducing uncertainty, remaining healthy, and staying optimistic) (Brashers et al., 2002). Yang and Kahlor (2013) separated the predictors of individuals' seeking and avoiding intention concerning information about climate change.

The RISP builds a relatively complicated depiction of the role of risk communication in individuals' potential behavioral change, aiming to disentangle the social, psychological, and communicative factors that motivate risk information seeking and processing in various contexts (Griffin et al., 1999a, 2004; Yang et al., 2010a,b). This model comprises two of TPB concepts—subjective norms and perceived control (information gathering capacity), while not containing two other TPB variables-attitude toward behavior and behavioral intention, which has been included in PRISM. In addition, PRISM focuses on information seeking rather than processing, which is also very essential for the RISP. These are the main differences between the RISP and PRISM. Compared to the RISP, the PRISM model has been validated by previous researchers and is a useful theoretical framework for predicting the intention to seek information in the situation of general health risks (Kahlor, 2010; Willoughby and Myrick, 2016), natural and environmental risks (Kahlor, 2007; Ho et al., 2014; Eastin et al., 2015; Kahlor et al., 2019) and specific diseases such as cancer (Hovick et al., 2014a), and the Zika virus (Hubner and Hovick, 2020). Therefore, this study is based on PRISM, which is viewed as more effective and appropriate in exploring our topic-information seeking and avoidance, rather than the former and aims to examine Chinese adults' COVID-19 risk information seeking and avoidance intentions during early stages of the COVID-19 outbreak.

### Risk Perception

Recent research has shown that an individual's cognitive appraisal of and affective response to a risk was an important predictor of his or her information seeking intention and perceived knowledge insufficiency across PRISM research in various risk communication environments (Eastin et al., 2015; Kahlor et al., 2019). When people are confronted with a particular risk, they evaluate their risk level and then determine whether they need more information on the topic. Specifically, when people sense a greater risk from a particular hazard, they will also have a greater emotional response to the issue (Loewenstein et al., 2001). As affective risk response related to these perceptions may be negative in nature due to the negative valence of risk perception (Griffin et al., 2004), increased risk perception could result in negative emotional response such as fear and anxiety (Witte, 1992; Turner et al., 2006), which would promote them to assess their existing information level and then decide whether they need to obtain additional information or process existing information more carefully (Yang, 2019). As COVID-19 has great disaster potential and fatal results, people will have high risk perception and strong emotional reactions, such as fear, anger, and anxiety.

When an individual feels worried, angry, scared, etc., he/she will positively search for related risk information in order to regain control over a situation, especially when their efficacy evaluation and threat evaluation are both high (Witte, 1994; Griffin et al., 2008). Moreover, the more scared (angry, or worried) they feel, less knowledgeable about their risk, the more actively seeking information, and the less avoidance. That is, affective risk response drives knowledge insufficiency. It, independent of a sense of information insufficiency, also motivates information seeking intention as emotions (including negative and positive emotions) stimulate the tendency to act and prepare for action (Frijda, 2004; Griffin et al., 2008; Kahlor, 2010). In addition, when people feel threatened or dangerous and feel powerless (low efficacy), they may turn to fear control and avoid further information to reduce their negative emotions (Witte, 1994). Thus, we predict that risk perception will be positively related to affective risk response (H1). We also propose that affective risk response will be positively related to knowledge insufficiency (H2) and COVID-19 information seeking intention (H3), while it will be negatively associated with information avoidance (H15).

### Beliefs-Based Variables

Within the RISP, informational subjective norms (ISN) are a powerful direct motivator for information seeking (Griffin et al., 2012a) and the direct and indirect impact of ISN on information seeking have been verified (Griffin et al., 2008). Within PRISM, this is also true (Kahlor, 2010; Hovick et al., 2014a,b). ISN indicate an individual's tendency to respond to social expectations that they should obtain adequate information to cope with a risky condition (Yang et al., 2014). That is, when individuals believe that people who are important to them want them to possess some knowledge regarding a risk issue, they are likely to more actively search for information (Yang and Kahlor, 2013). In the case of COVID-19, people who believe that information seeking is a normative behavior, will want more information than they currently have (Kahlor, 2010). Therefore, we expect that ISN will be positively related to information seeking intention (H4), but negatively related to information avoidance (H16). In addition, it is expected that ISN will be positively related to perceived knowledge (H5) and perceived knowledge insufficiency (H6).

According to Ajzen (1991), attitude toward seeking information within PRISM, rooted in TPB, refers to an individual's assessment of whether information seeking would be beneficial to him or her. If an individual perceives that performing the behavior (information seeking) would be in his or her favor, the individual will be more likely to perform the behavior, while less likely to avoid it, just for this reason that attitude toward information seeking is negatively related to information avoidance (Yang and Kahlor, 2013; Howell et al., 2016). That is, when individuals have more positive attitudes toward seeking risk information, they are less likely to avoid it. Other research has shown that implicit attitudes about learning health information predicted risk information avoidance (Howell et al., 2016). Thus, we put forward the following hypotheses, which are supported by previous studies (Kahlor, 2010; Eastin et al., 2015; Kahlor et al., 2019; Hubner and Hovick, 2020): attitude toward seeking information will be positively related to seeking intention (H7), but negatively related to information avoidance (H17). In addition, we expect attitude toward information seeking to be positively related to perceived knowledge (H8) and perceived knowledge insufficiency (H9).

Within PRISM, perceived seeking control (PSC) captures both perceived se-lf-efficacy and the perceived controllability of the behavior (Kahlor, 2010), which is akin to the concept of perceived information gathering capacity in the RISP. That is, it is more challenging for members of the public with lower ability to choose a trustworthy information source and to discover the most valuable information to help them make risk decisions (Yang et al., 2014). Past studies have shown mixed results (Kahlor, 2007; Yang, 2012). Currently, people have easier access to risk-related information involving the ways the novel coronavirus is transmitted and ways to protect themselves due to the popularity of the Internet and the government's vigorous publicity. This appears to make individuals sense that their ability to search for information has increased and they have a high level of knowledge about COVID-19. Thus, according to PRISM, we expect that PSC will be positively related to information seeking intention (H10), perceived knowledge (H11) and perceived knowledge insufficiency (H12), but negatively related to information avoidance (H18) proposed by Yang and Kahlor (2013).

### Perceived Knowledge and Knowledge Insufficiency

The RISP model shows that individuals' psychological need for knowledge sufficiency termed knowledge insufficiency, which is one of the more notable concepts of PRISM, primarily motivates actively seeking and systematically processing risk information. In the RISP, knowledge insufficiency concept is based on Eagly and Chaiken's Heuristic Systematic Model, especially the “accuracy” motivation which is part of the drive for sufficiency in judgmental confidence in HSM. There is a gap between an individual's current level of understanding and the desired level of understanding, which should be related to seeking more information hardly to achieve processing goals (Eagly and Chaiken, 1993). The concept of knowledge insufficiency in the RISP is based on the above assumption and shows that information seeking is driven by the gap between what one currently knows and the knowledge needed to fully understand a given topic (Griffin et al., 2004, 2012b; Kahlor, 2010). A person will continue to actively process related information until they have achieved the depth or breadth of understanding that they conceive to be sufficient (Eagly and Chaiken, 1993). W-hen applied in the RISP, respondents are essentially asked how much more Knowledge they need in order to deal adequately with the risk in their own lives, that is, ho-w much would be sufficient, which is used for reference in PRISM. Moreover, in the latter model, perceived knowledge insufficiency is driven by perceived knowledge, affective risk response, subjective norms, attitudes, and perceived behavioral control.

**Figure 1:**
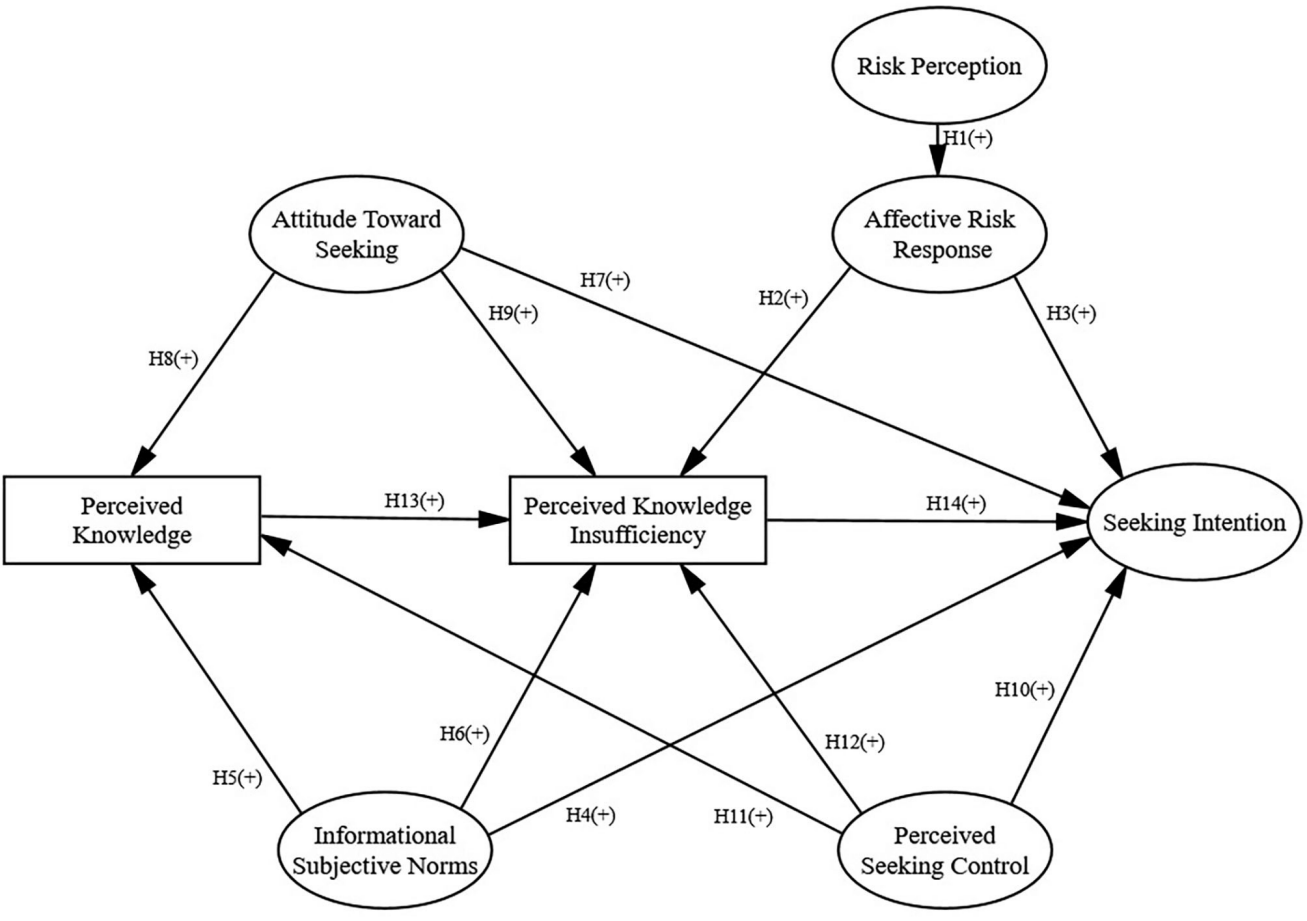
The study's conceptual model based on PRISM, with seeking intention as the endogenous variable.

**Figure 2:**
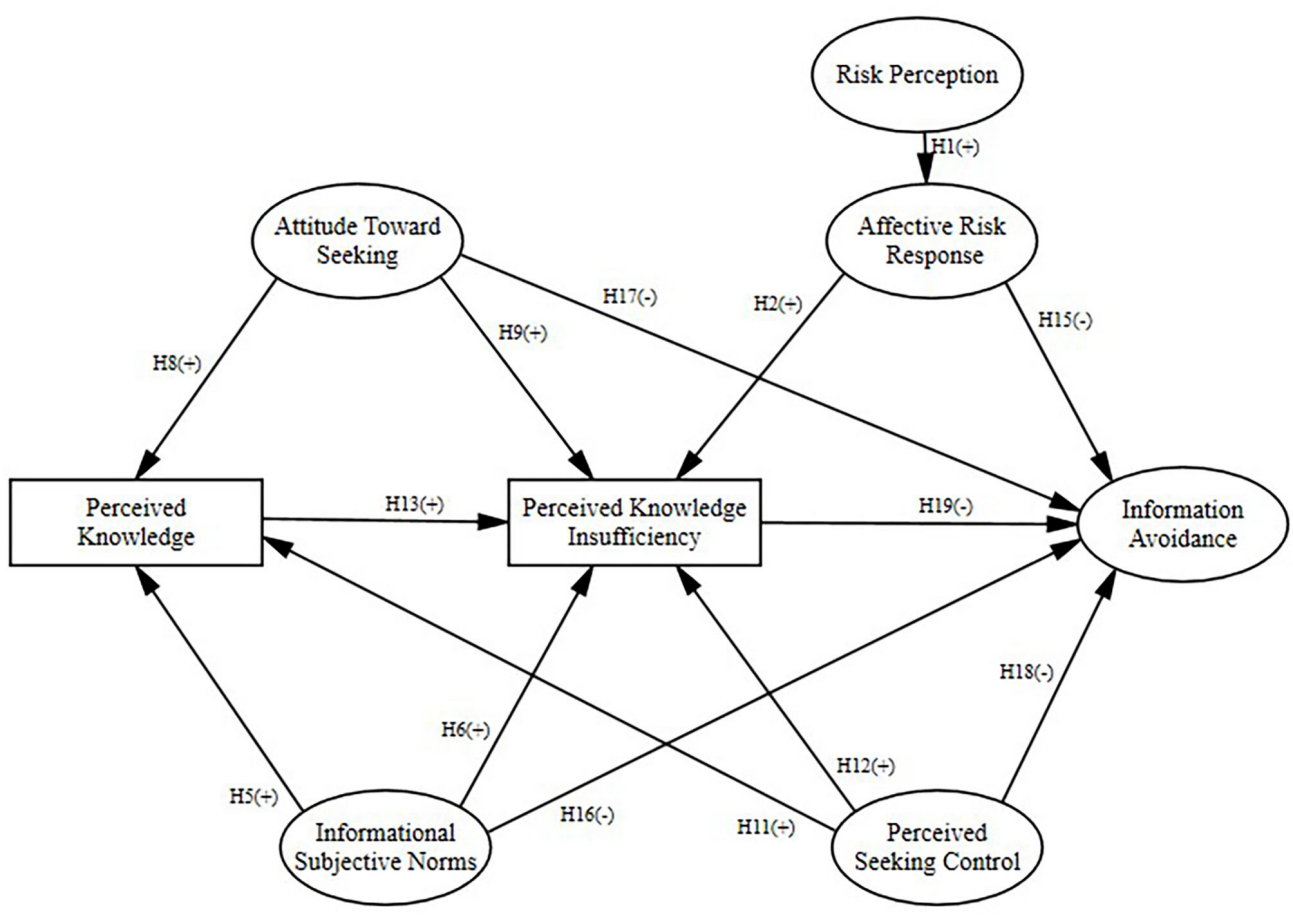
The study's conceptual model based on PRISM, with information avoidance as the endogenous variable.

Among PRISM studies, full or partial support for a relationship between perceived knowledge insufficiency and information-seeking intent has been found in previous studies (Kahlor, 2007; Hovick et al., 2014a) but has not been found in other studies (Kahlor, 2010; Hubner and Hovick, 2020). One RISP-related study suggests that the more perceived knowledge insufficiency, the less likely information avoidance (Dunwoody and Griffin, 2015). Therefore, it is hypothesized that the more knowledge people have, the more knowledge insufficiency they perceive (H13), which increases their intention to seek information (H14), and decreases information avoidance (H19). All the above hypotheses are shown in Figures 1 and 2, respectively.

## Methods

### Sample and Procedure

Data were gathered from an online survey in Wuhan city, Hubei Province in China. The respondents were told that the purpose of this survey was only for research, that participation was voluntary and that their responses were confidential in a cover letter attached to each questionnaire. The survey included items concerning risk perception, affective risk response, informational subjective norms, perceived seeking control, attitude toward seeking, perceived knowledge, perceived knowledge insufficiency, information seeking intention, information avoidance, and demographic information. Before collecting the research data, we contacted HR directors in each school and asked them to randomly select grades and classes in their school. Respondents were invited to take the survey and were eligible for a several-yuan (equivalent to ranging from 0.31 USD to 1.40 USD) prize after completing the questionnaire.

A total of 1,031 Chinese adults from eight schools including elementary, middle and high schools, and universities, participated in this survey online from March 3 to 5, 2020. The survey subjects included both university students and residents in Wuhan, who were the parents or teachers of students from the above schools. After deleting questionable and conflicting data, our effective sample size was 822. As shown in Table 1, the sample was predominantly female (63.5%) and living in cities (69.2%). Almost 43 percent of respondents were 18–25 years old, followed by those 41–50 years old (26.3%). Over 90 percent had more than a high school education. Nearly 60% lived in Hubei Province because among our survey respondents, university students studying in Wuhan and some Wuhan residents lived in their hometowns outside Hubei province when we conducted this survey. A total of 37.4% of the participants reported that their monthly household income per capita was less than RMB ¥5000, followed by RMB ¥5001–10000, accounting for 32.2%.

**Table 1:** Descriptive statistics analysis for demographic information.

**Demographic traits**	**Frequency**	**Percentage (%)**
**Gender**		
Female	522	63.5
Male	300	36.5
**Age**		
18–25	353	42.9
26–30	96	11.7
31–40	128	15.6
41–50	216	26.3
51–60	25	3.0
Over 60	4	0.5
**Education**		
Middle school or less	30	3.7
High school or equivalent	46	5.6
College	108	13.1
Undergraduate	374	45.5
Master or above	264	32.1
**Average household's monthly income**		
Less than or equal to 5000	307	37.4
5001–10000	265	32.2
10001–15000	135	16.4
15001–20000	53	6.5
More than 20000	62	7.5
**Current location**		
Wuhan city	304	37.0
Other cities in Hubei Province	185	22.5
Other cities except Hubei Province	333	40.5
**City or rural**		
City	569	69.2
Rural	253	30.8

### Measures

All original scales were developed in English and finally presented in Chinese. We employed back-translation procedures to assure the reliability and validity of the scales.

### Perceived Knowledge

Based on past research (Kahlor, 2010), participants were asked “How much knowledge do you have about your own risk of being infected with COVID-19?” to assess perceived knowledge of COVID-19. In order to be consistent with other measures, the item was rescaled from a 0–100 scale to a 0–10 scale.

### Perceived Knowledge Insufficiency

Knowledge insufficiency was conceptualized as the gap between knowledge owned and knowledge needed (Griffin et al., 2004; Hovick et al., 2014a). It is worth noting that the knowledge here specifically refers to the knowledge related to people's risk of contracting COVID-19. Respondents were asked “How much do you need to know about your risk of infection from COVID-19?” The item was rescaled from a 0–100 scale to a 0–10 scale for consistency with other measures.

### Risk Perception

In both interpersonal and mediated communication studies, risk perception often focuses on the perceived likelihood and severity of an event (Kasperson et al., 1988). Thus, risk perception was measured by means of two items. These items assessed the public's perception of likelihood and seriousness of future illness, which were measured on a scale ranging from 1 (not at all likely/serious) to 10 (very likely/serious) scale.

### Affective Risk Response

Based on previous work (Yang and Kahlor, 2013; Hovick et al., 2014a), we asked participants whether they felt worried, scared, or angry as well as an overall measure of whether they had negative feelings about the risk of COVID-19 to measure their negative affective response to the risk (Cronbach's α = 0.84).

### Attitude Toward Seeking

Attitude toward seeking is a general assessment (including instrumental and experiential evaluation) of performing the behavior of–seeking information about COVID-19 risk (Kahlor, 2007) (Cronbach's α = 0.96).

### Informational Subjective Norms

Five items measuring informational subjective norms, which were consistent with Ajzen's (2002) conceptualization, were adapted from past studies (Ajzen and Fishbein, 2005) on a 1 (strongly disagree) to 5 (strongly agree) scale. Two dimensions (injunctive norms and descriptive norms) were included (Cronbach's α = 0.91).

### Perceived Seeking Control

Perceived seeking control was measured by four items on a scale of 1 (strongly disagree) to 5 (strongly agree) based on Ajzen (2002) and Kahlor (2010) (Cronbach's α = 0.91).

### Seeking Intention

Adapted from previous studies (Kahlor, 2010; Yang and Kahlor, 2013), information seeking intention was measured on a sale of 1 (strongly disagree) to 5 (strongly agree) (Cronbach's α = 0.94). Four items were contained.

### Information Avoidance

Information avoidance was measured by five items on a 1 (strongly disagree) to 5 (strongly agree) scale (Cronbach's α = 0.96). These measures were adapted from previous research (Yang and Kahlor, 2013).

All measurement items are outlined in Table 2.

**Table 2:** The items of constructs of the questionnaire.

**Variable**	**Measure**	**Source**
Perceived knowledge	How much knowledge do you have about your own risk of being infected with COVID-19?	Kahlor, 2010
Perceived knowledge insufficiency	How much do you need to know about your risk of infection with COVID-19?	Griffin et al., 1999b; Hovick et al., 2014a
Risk perception	How likely is it that you will become infected with COVID-19? If you were to become infected with COVID-19, how severe would it be?	Hovick et al., 2014a
Affective risk response	How much of the following do you feel about COVID-19? Not worried…Very worried Not scared…Very scared Not angry…Very angry I have negative feelings about COVID-19.	Yang and Kahlor, 2013; Hovick et al., 2014a
Attitude toward seeking	To consider words that can be used to describe information seeking about your risk for COVID-19… Worthless…Valuable Bad…Good Harmful…Beneficial Not helpful…Helpful	Kahlor, 2007
Informational subjective norms	Injunctive norms: It is expected of me that I seek information about my COVID-19 risk. Most people who are important to me think that I should seek information about my COVID-19 risk. Others expect me to seek information about my COVID-19 risk. My family expects me to seek information about my COVID-19 risk. Descriptive norms: People in my life whose opinions I value seek information about their own COVID-19 risk.	Ajzen and Fishbein, 2005
Perceived seeking control	I know where to look for information about my own COVID-19 risk. I know how to search for information about my COVID-19 risk. When it comes to information about my COVID-19 risk, I know how to separate fact from fiction. I can readily access all the information about COVID-19 risk that I need.	Ajzen, 2002; Kahlor, 2010
Seeking intention	I plan to seek information about my COVID-19 risk in the near future. I will try and seek information about my COVID-19 risk in the near future. I intend to find more information about my COVID-19 risk soon. I will look for information about my COVID-19 risk in the near future.	Kahlor, 2010; Yang and Kahlor, 2013
Information avoidance	I avoid information about my COVID-19 risk. I refuse to listen to information about my COVID-19 risk. I ignore information about my COVID-19 risk. I ignore the information about my COVID-19 risk. When it comes to the risk of COVID-19, I don't want to know more.	Yang and Kahlor, 2013

Both models were tested by latent-variable structural equation modeling with the statistical package AMOS 24.0. The method of estimation for unknown parameters was the maximum likelihood procedure.

## Results

Among the respondents, 91.6% reported that they washed their hands with soap or hand sanitizers more frequently during the pandemic than before and 65% avoided going to the hospital even though they were ill. The most common channel for people to obtain information on COVID-19 risk was the WeChat public platform, followed by microblogs, TV, web portals, news and information apps, and search engines. The information of greatest concern was the latest progress of the pandemic (e.g., maps of the COVID-19 pandemic, progress in vaccine research, and the latest news on the pandemic in Wuhan), followed by pandemic prevention knowledge and relevant government policies. Based on the above, we can observe that the public paid attention to some information about COVID-19, including the method of transmission, and was willing to take action to protect themselves and their families from infection.

Table 3 shows spearman correlations, means, and standard deviations for the model variables.

**Table 3:** Spearman correlations, means, and standard deviations for model variables (*N* = 822).

	**1**	**2**	**3**	**4**	**5**	**6**	**7**	**8**	**9**	**M**	**SD**
1. Attitude toward seeking	1									5.69	1.27
2. Informational subjective norms	0.44^**^	1								3.87	0.79
3. Perceived seeking control	0.31^**^	0.54^**^	1							3.82	0.81
4. Risk perception	−0.001	0.05	0.08^*^	1						19.29	18.84
5. Affective risk response	0.05	0.12^**^	0.15^**^	0.22^**^	1					5.51	2.13
6. Perceived knowledge	0.25^**^	0.26^**^	0.23^**^	−0.03	−0.03	1				8.79	1.78
7. Perceived knowledge insufficiency	0.26^**^	0.27^**^	0.19^**^	0.04	0.04	0.54^**^	1			9.14	1.79
8. Seeking intention	0.37^**^	0.66^**^	0.56^**^	0.06	0.17^**^	0.22^**^	0.25^**^	1		3.70	0.84
9. Information avoidance	−0.40^**^	−0.40^**^	−0.27^**^	0.05	0.01	−0.25^**^	−0.29^**^	−0.40^**^	1	1.82	0.82

** *P < 0.01*,

* *P < 0.05*.

### Seeking Intention

The PRISM model was tested with information seeking intention as the dependent variable. We first tested a measurement model including 5 factors and 21 indicators. The overall fit of the measurement model was excellent [χ2(179) = 647.02 (*p* < 0.01), RMSEA = 0.06 (90% CI = (0.052, 0.061)), CFI = 0.97, SRMR = 0.05] and all standardized factor loadings were greater than or equal to 0.63.

**Figure 3:**
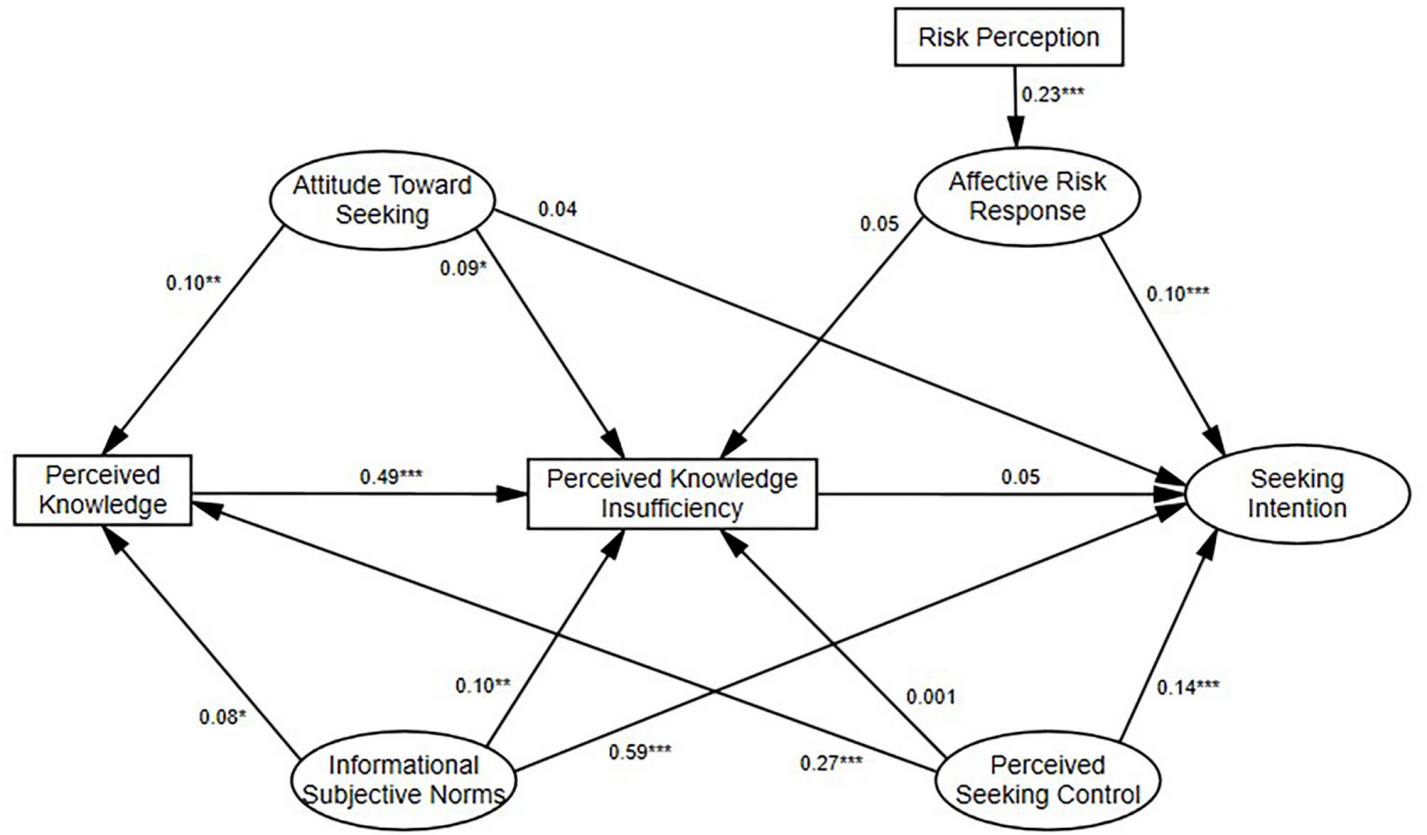
Standardized path coefficients for PRISM predicting COVID-19 seeking intention. ***P < 0.001, **P < 0.01, *P < 0.05.

We then added the proposed structural paths to test Hypotheses H1–14. The model fit the data well [χ2(273) = 850.11 (*p* < 0.01), RMSEA = 0.05 (90% CI = (0.047, 0.055)), CFI = 0.96, SRMR = 0.05] and explained 32% of the variance in knowledge insufficiency and 51% of the variance in seeking intention. The results of the hypotheses tests (standardized path coefficient) are shown in Figure 3.

Most study hypotheses were confirmed, except for the relationships between affective risk response and knowledge insufficiency (Hypothesis 2), subjective norms and knowledge (Hypothesis 5), perceived seeking control and knowledge insufficiency (Hypothesis 12), attitude toward seeking and seeking intention (Hypothesis 7), and perceived knowledge insufficiency and seeking intention (Hypothesis 14).

### Information Avoidance

We then used PRISM to assess COVID-19-related information avoidance. A second measurement model was tested with 5 factors and 22 indicators. The measurement model's overall fit was good [χ^2^(199) = 791.19 (*p* < 0.01), RMSEA = 0.06 (90% CI = (0.056, 0.065)), CFI = 0.96, SRMR = 0.05], and all standardized factor loadings were >0.63.

**Figure 4:**
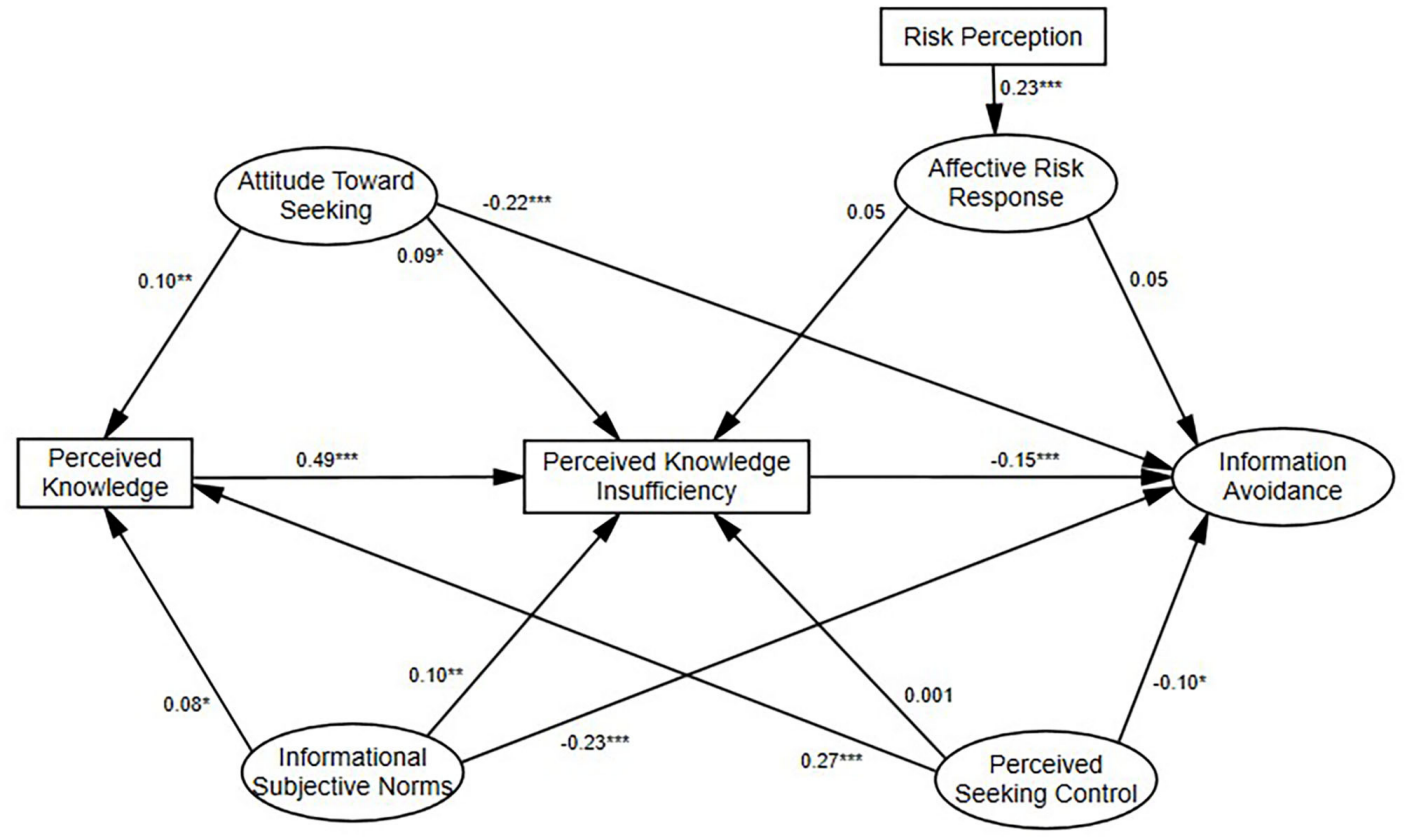
Standardized path coefficients for PRISM predicting COVID-19 information avoidance. ***P < 0.001, **P < 0.01, *P < 0.05.

After the presented structural paths were added to the model (Hypotheses 1–19 except for 3,4,7,10, and 14), the structural model fit the data well [χ^2^(298) = 963.54 (*p* < 0.01), RMSEA = 0.05 (90% CI = (0.048, 0.056)), CFI = 0.96, SRMR = 0.05]. This model explained 26% of the variance in information avoidance. As shown in Figure 4, attitude toward seeking, subjective norms, perceived knowledge insufficiency, and perceived seeking control were negatively related to information avoidance (H16–19 supported). Most of the other hypotheses were also supported. Especially, knowledge was positively related to knowledge insufficiency (H13 supported).

## Discussion

We examined PRISM in the situation of individuals' COVID-19 risk information seeking behavior and offered support for the applicability of the PRISM model to forecast the intentions of information seeking and avoidance during the pandemic.

Intention seeking about COVID-19 risk was directly influenced by affective risk response, ISN and PSC. However, information avoidance was primarily driven by attitude toward seeking, ISN, PSC, and knowledge insufficiency. Among these predictors, our findings indicate that ISN are the strongest predictor of information seeking and avoidance. People who pay attention to others' expectations regarding their behavior may prefer to seek information, and may be less willing to avoid related information. Furthermore, subjective norms were positively related to knowledge insufficiency. Therefore, when designing risk communication strategies targeted at the public, the focus should be informational subjective norms.

Consistent with prior studies (Kahlor, 2010; Hovick et al., 2014a), risk perception was positively related to affective risk response, which was associated with the intention to seek information but was not associated with information avoidance. That is, higher COVID-19 risk perception is associated with a more negative response, which leads to greater seeking intention. When people feel more worried, scared, or angry, they want to seek more information to ensure that they can take measures to avoid infection (instrumental value offered by information) and relieve their tension, anxiety, and panic (emotional value offered by information). However, affective risk response is not proven to be a powerful driver of information seeking. Compared with ISN and PSC, affective risk response plays a more marginal role in determining seeking information. This issue may be related to our survey time. At the beginning of March, the pandemic situation in China, especially in Wuhan City, was undergoing positive changes after the Chinese government adopted timely and effective prevention and control measures. Thus, people's negative response to COVID-19 risk, such as anxiety and panic, was relieved to some extent, and its impact on their information decision-making behavior was reduced. These are our speculations. Future research should explore the relationship between positive and negative affective response and information seeking as well as the relationship between various affective response and information avoidance.

Furthermore, attitude toward information seeking was positively related to both knowledge and knowledge insufficiency but negatively related to information avoidance. People who hold a favorable attitude toward COVID-19 information seeking are more likely to perceive that they have a greater knowledge base about this topic, perceive the need for more knowledge and are less likely to avoid new information. A relationship was not found between attitude toward seeking and information seeking, which needs further exploration.

In addition, PSC was positively associated with information seeking but negatively associated with information avoidance. People with greater perceived information-seeking capacity (information accessibility and understandability) are more likely to seek COVID-19 information and less likely to avoid new information. As we expected, PSC played a relatively small role, especially in driving information seeking and avoidance. In addition, PSC was a significant predictor of perceived knowledge. That is, people with a higher capacity for information seeking perceive that they have a greater amount of related knowledge.

As we conjectured, perceived knowledge was significantly related to perceived knowledge insufficiency in the context of COVID-19. People who perceived that they possessed greater knowledge of COVID-19 were more likely to report greater knowledge insufficiency (i.e., the perceived need for more knowledge or information). In addition, as Hubner and Hovick (2020) mentioned, the lack of the relationship between perceived knowledge insufficiency and information seeking intention, which is consistent with Kahlor's results (Kahlor, 2010), implies that people who perceived higher knowledge insufficiency may not result in more active information seeking, which does not necessarily reduce uncertainty in the context fraught with uncertainty and limited related information.

## Study Limitations

First, one of the limitations is related to survey time. We collected data at a point in time during the pandemic, which made us unable to determine whether there were different relationships between model variables within PRISM at different stages of the pandemic. Future studies can conduct several investigations at different times of the pandemic, such as the early stage, peak stage, and aggravation stage, to make such a comparison. Then, the other limitation is that the perceived source or channel characteristics are not considered within PRISM, while other models such as the RISP treat it as an important predictor to the intention to search for risk information (Johnson and Meischke, 1993; Griffin et al., 1999a; Afifi and Weiner, 2004). Therefore, beliefs in information sources deserve more attention from PRISM researchers.

## Conclusion

Given that PRISM is a theoretical framework that demonstrates the importance of the public's information sufficiency, risk perception, subjective norms, and self-efficacy as predictors of risk information seeking behavior, we draw on this model to predict not only the public's information seeking but also their information avoidance during the COVID-19 outbreak. Our results provide theoretical and empirical insights into those predictors and offer empirical support for the assertion of Kahlor (2010)—PRISM performed better than the TPB and RISP models in demonstrating people's information seeking and avoidance intentions. Information seeking is mainly directed by ISN and PSC, with affective risk response playing only a marginal role. We also explored the predictors of information avoidance, which included ISN, attitude toward seeking, knowledge insufficiency and PSC. Among them, the first two played a greater role in driving information avoidance. Particularly, ISN were the strongest predictor of both information seeking and avoidance. Therefore, ISN should be the focus when designing risk information communication strategies. People may be driven by information that emphasizes perceived pressure from the others to become more active information searchers (Hovick et al., 2014a). Future research should further explore what factors may influence informational subjective norms and how to highlight their role in designing risk communication strategies and carrying out communication practices.

Additionally, the public's positive attitude toward information seeking should be fostered and their emotions, especially negative emotions should be monitored to carry out targeted COVID-19 risk communication and improve its efficiency and effectiveness. Specifically, information from sources trusted by the public should be released through credible channels that are easily accessible to the public, such as the WeChat public platform, microblogs, TV, web portals (e.g., sohu.com), and news and information apps. In addition, the information of greatest concern to the public should be updated and released in a timely manner. Such information includes the latest progress of the pandemic, knowledge of pandemic prevention and control, and relevant government policies, which should be clear, accurate, and useful. Especially, we should pay special attention to the release of the opinions of some leaders (e.g., Academician Nanshan Zhong and Lanjuan Li, who are highly respected and trusted by the public in China), which is very helpful to relieve people's panic, especially during the severe period of the pandemic. Through the above measures, the supply of knowledge can be increased to meet people's urgent information needs so that they can strengthen their self-protection and relieve their anxiety and panic to actively cooperate with government initiatives.

## Data Availability Statement

The raw data supporting the conclusions of this article will be made available by the authors, without undue reservation, to any qualified researcher.

## Ethics Statement

The studies involving human participants were reviewed and approved by the ethics commitment of Huazhong Agricultural University. The patients/participants provided their written informed consent to participate in this study.

## Author Contributions

ML and YC implemented the questionnaire and analyzed the results. ML drafted the manuscript. DS made some suggestions. TY provided the critical feedback. All authors contributed to research design, data collection, and reviewing.

## Conflict of Interest

The authors declare that the research was conducted in the absence of any commercial or financial relationships that could be construed as a potential conflict of interest.
